# Lixisenatide in type 1 diabetes: A randomised control trial of the effect of lixisenatide on post‐meal glucose excursions and glucagon in type 1 diabetes patients

**DOI:** 10.1002/edm2.130

**Published:** 2020-06-12

**Authors:** Chitrabhanu Ballav, Archana Dhere, Irene Kennedy, Olorunsola F. Agbaje, Sarah White, Rachel Franklin, Bolette Hartmann, Jens J. Holst, Rury R. Holman, Katharine R. Owen

**Affiliations:** ^1^ Oxford Centre for Diabetes Endocrinology and Metabolism University of Oxford Churchill Hospital Oxford UK; ^2^ Diabetes Trials Unit University of Oxford Oxford UK; ^3^ Oxford NIHR Biomedical Research Centre Oxford University Hospitals Oxford UK; ^4^ NNF Center for Basic Metabolic Research and Department of Biomedical Sciences University of Copenhagen Copenhagen Denmark

**Keywords:** GLP1 analogue, lixisenatide, type 1 diabetes

## Abstract

**Aims:**

The GLP1 agonist lixisenatide is glucagonostatic and reduces post‐prandial blood glucose (PPBG) in type 2 diabetes. This study investigates its impact in type 1 diabetes (T1D).

**Methods:**

In a blinded, crossover trial, 25 patients with T1D were randomised to 4 weeks adjunctive treatment with lixisenatide (L) or placebo (P), with a 4‐week washout period. The primary outcome was percentage of 3 hours PPBG in target (4‐10 mmol/L) assessed by CGM before and after treatment. Participants also underwent post‐treatment standardised mixed meal test (MMT, n = 25) and hyperinsulinaemic hypoglycaemic clamp (n = 15).

**Results:**

PPBG CGM readings in target were similar between L vs P (Mean % ± SE, breakfast 45.4 ± 6.0 vs 44.3 ± 6.0, *P* = .48, lunch 45.5 ± 5.8 vs 50.6 ± 5.3, *P* = .27 and dinner 43.0 ± 6.7 vs 47.7 ± 5.6, *P* = .30). HbA1C was similar between L vs P (64.7 ± 1.6 vs 64.1 ± 1.6 mmol/mol, *P* = .30). Prandial insulin fell after lixisenatide (dose change −0.7 ± 0.6 vs +2.4 ± 0.7 units/d, *P* = .004), but basal insulin dose was similar between groups. The post‐MMT glucose area under the curve (AUC) was lower with L than P (392.0 ± 167.7 vs 628.1 ± 132.5 mmol/L × min, *P* < .001), as was the corresponding glucagon AUC (140.0 ± 110.0 vs 304.2 ± 148.2 nmol/L × min, *P* < .001). Glucagon and counter‐regulatory hormone values at a blood glucose of 2.4 mmol/L during the hypoglycaemic clamp were similar between L and P.

**Conclusion:**

In T1D, PPBG values were not altered by adjunctive lixisenatide although prandial insulin dose fell. Glucose and glucagon level during an MMT were significantly lower after lixisenatide, without affecting counter‐regulatory response during hypoglycaemia.

## Graphical abstract

In type 1 diabetes patients, post‐prandial blood glucose values were not altered by adjunctive lixisenatide although prandial insulin dose fell. Lixisenatide significantly lowered glucose and glucagon level after standardised mixed meal, without affecting counter‐regulatory response during hypoglycaemia.

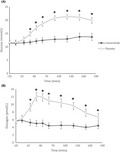

## 1 INTRODUCTION

Only 30% of patients with type 1 Diabetes (T1D) achieve a glycaemic goal of HbA1C <7.5% (58 mmol/mol).^1^ Post‐prandial hyperglycaemia is common in all types of diabetes and may have a role in overall glycaemic control.^2^ It is generally agreed that reducing glucose excursion after meal improves overall glycaemic control,^3^ although recent reviews have cast a doubt on the effect of short glucose variability on long‐term diabetes complications.^4^

Glucagon levels are physiologically suppressed at high plasma glucose concentrations. In the absence of diabetes and in T2D, glucagon level is found to be higher after oral glucose compared with isoglycaemic intravenous glucose infusion, although more insulin is secreted when glucose is administered by the oral route. This has been attributed to the glucagonotropic effect of GIP.^5^ In people with T1D with no detectable β cell function, paradoxically high levels of glucagon have been noticed after 50 g oral glucose compared with isoglycaemic glucose infusion.^6^ Post‐prandial hyperglucagonaemia following a MMT has been found to worsen progressively in the first year after a new diagnosis of T1D, as C‐peptide levels decline.^7^, ^8^ Nonsuppression of glucagon contributes to post‐prandial hyperglycaemia in T1D and therefore may have a role in treatment.

The short‐acting exendin‐based glucagon‐like peptide‐1 (GLP1) receptor agonist lixisenatide reduces post‐prandial hyperglycaemia by suppressing glucagon, and by slowing gastric emptying^9^ in T2D. In this study, we investigate its effect in patients with T1D.

## 2 MATERIALS AND METHODS

### 2.1 Study design

**FIGURE 1:**
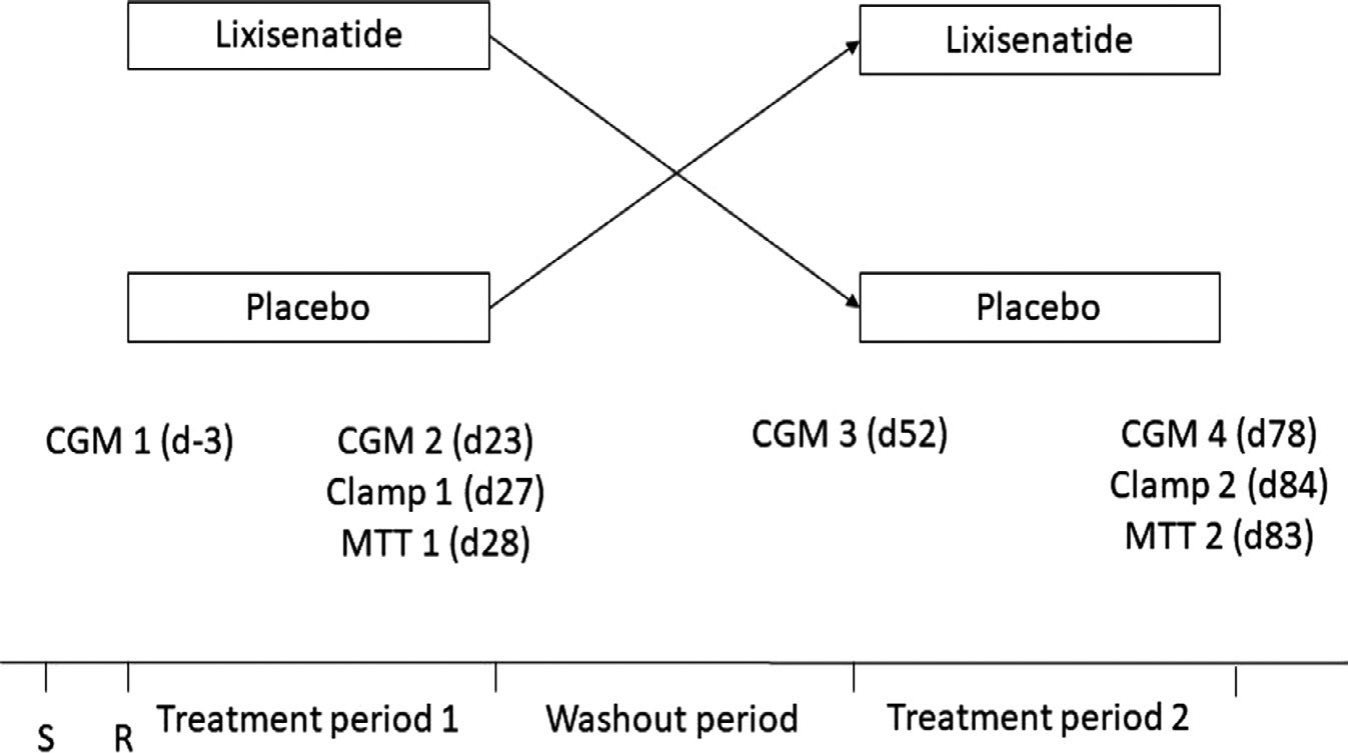
Trial design

A single centre, double blind, placebo‐controlled crossover trial was performed (Figure 1). Patients were enrolled from the outpatient clinic at the Oxford Centre for Diabetes Endocrinology and Metabolism. The study was approved by the clinical ethics committee of UK, registered with ISCRTN (No. 00290196), performed according to Good Clinical Practice and and externally monitored. Following informed consent, participants were randomised by a computer‐generated programme to receive treatment for 4 weeks with lixisenatide (10 µg/d titrated up to 20 µg/d in 2 weeks if tolerated) or placebo in the morning, along with their usual insulin, in random order with a washout period of 4 weeks between treatments. During the treatment period, the usual dose of insulin was reduced (−20% basal insulin, −50% bolus at breakfast, and −20% bolus at lunch). Participants were advised on insulin titration to maintain blood glucose between 6 and 9 mmol/L, guided by investigators. They received a phone call at the beginning of week 3 to check on side effects, and to advise titration of the trial drug to 20 µg/d. They were advised to follow their usual daily routine.

Participants underwent continuous glucose monitoring (CGM) with Navigator, Abbott Diabetes Care for 3‐5 days at the beginning and before the end of the treatment period. Standardised advice on calibration and hypoglycaemia avoidance were provided. Patients maintained a diary of meal times during this period, and data for first 72 hours of CGM were used for analysis of the primary end‐point, defined as proportion of CGM readings between 4 and 10 mmol/L in the 3 hours post‐prandial period following the three major meals (breakfast, lunch and evening meal). At the final 2 days of treatment period, patients were given the option to have a standardised mixed‐meal test (MMT) and a hyperinsulinaemic‐hypoglycaemic clamp (clamp).

Secondary end‐points included comparison between changes in insulin doses between treatment groups, and end‐points after MMT and Clamp. The MMT (240 mL Fortisip liquid containing 18.4 g carbohydrate/100 mL) was performed in the penultimate day of each treatment period, in the morning after overnight fast, and 20 minutes after their blinded study medication. No prandial insulin was provided. Glucose and glucagon concentrations were measured at baseline and every 30 minutes for 2 hours between treatment groups.

The clamp was performed in the final day of the treatment period, in the morning after overnight fast, and 20 minutes after their blinded study medication. Participants with a high blood glucose level (10‐15 mmol/L) were given intravenous insulin to reduce their blood glucose to 10 mmol/L. Participants received a primed, continuous infusion of insulin (Actrapid) at 3.0 mU/kg/min from 0 to 4 minutes, 2.5 mU/kg/min from 4 to 7 minutes, 2.0 mU/kg/min from 7 to 10 minutes and 1 mU/kg/min thereafter, with 20% glucose infused at a variable rate to achieve three steps of glycaemic plateau of 7.5 mmol/L of glucose (time 0‐45 minutes), the second, euglycaemic at 5.0 mmol/L (to reach 5.0 mmol/L between 45 and 90 minutes and maintain a plateau of 5.0 mmol/L for 90‐135 minutes) and the third, hypoglycaemic at 2.5 mmol/L (to reach 2.5 mmol/L between 135 and 180 minutes and maintain a plateau of 2.5 mmol/L for 180‐225 minutes). After the hypoglycaemic step, the insulin infusion was discontinued and glucose was infused if necessary to allow recovery of blood glucose to more than 4 mmol/L at least on two readings. The rates of glucose infusion were adjusted according to established algorithms, guided by real‐time glucose measurements taken at the bed side (1 mL/measurement) every 5 minutes and measured by the glucose dehydrogenase technique using a HemoCue device (HemoCue). The procedure was performed with patients in supine position, and samples for glucose and glucagon were collected every 15 minutes for 225 minutes. Other counter‐regulatory hormones (adrenaline, noradrenaline, cortisol and pancreatic polypeptide) were sampled during the hypoglycaemic phase, between 180 and 225 minutes.

### 2.2 Laboratory measurements

Electrolytes and glucose concentrations were measured at the CPA‐accredited Oxford University Hospitals NHS Trust biochemistry laboratory (ADVIA 2400 general chemistry analyser). C‐peptide and cortisol were analysed using chemiluminescence immunoassay (ADVIA Centaur analyser using Siemens Healthcare Diagnostics Ltd.). HbA1C was measured using ion‐exchange chromatography (Menarini 8160 Diagnostics). Adrenaline, noradrenaline and pancreatic polypeptide were measured using ELISA. Glucagon was measured using a sandwich ELISA, which uses antibodies to both C‐ and N‐ terminal antiglucagon antibodies, and eliminates cross‐reactivity with elongated or truncated forms of glucagon peptide.^10^

### 2.3 Statistical analysis

The sample size was calculated based on the percentage of readings between 4 and 10 mmol/L derived using CGM for 3 hours post‐prandial period. As no data were available for lixisenatide in any previous cohort, best estimates derived from similar data sets^11^ have been used for our power calculation. The mean of the primary outcome and the within‐patient standard deviation in the targeted population was estimated to be 55.3% and 13.7%, respectively, using data published from a randomised controlled trial of 91 insulin‐requiring patients (75 with type 1 and 16 with type 2 diabetes). Using a significance level (alpha value) of .05 and allowing a 10% drop out rate, a sample size of 30 patients would provide a 90% power to detect a 15% increase in the primary outcome, defined as a beneficial effect of lixisenatide on post‐prandial glucose levels of CGM.

Data are expressed in means ± SD or SEM. Baseline data are represented as mean (SD), when values are compared between treatment and placebo arms mean (SEM) has been used. Normally distributed data were compared using the Student's *t* test (paired within and unpaired between groups). Nonparametric data were compared with Mann‐Whitney *U* test between groups, and Wilcoxon test for paired differences within groups. Statistical significance was *P* < .05. Statistical analysis has been done on SPSS 22.

## 3 RESULTS

### 3.1 Subjects and effect on baseline characteristics

**FIGURE 2:**
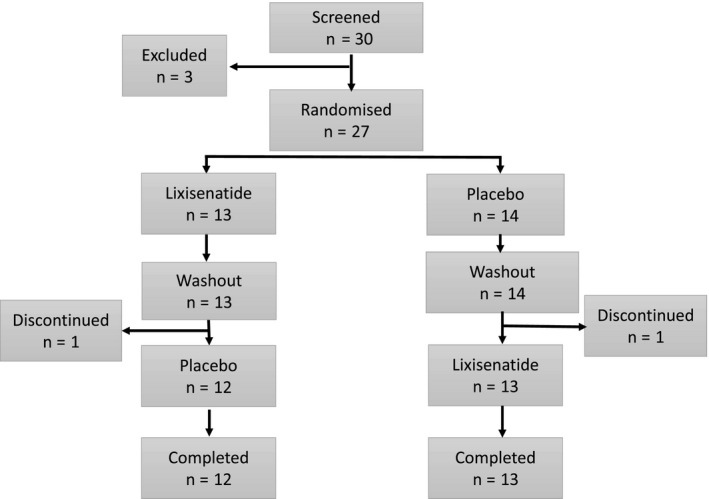
Screening, randomisation and retention

Patients were recruited from outpatient clinics of Oxford University Hospitals NHS Trust by the investigators, from January 2014 to August 2016. Recruitment was closed after 30 subjects had been screened, and 27 were randomised (Figure 2). A total of 25 participants completed the trial as two stopped after the first treatment period (one who became pregnant, and one who withdrew consent). Baseline characteristics of the participants are summarised in Table 1.

**TABLE 1:** Key baseline characteristics in mean (SD)

Baseline characteristics	Mean (SD)
Females	13
Age (y)	44 (2.5)
Duration of diabetes (y)	18.6 (14.2)
Insulin (basal U/d)	27.32 (15.1)
Insulin (prandial U/d)	18.28 (14.3)
FPG (mmol/L)	10.2 (4.5)
C‐peptide (nmol/L)	0.03 (0.04)
Creatinine (µmol/L)	70.46 (15.81)
eGFR (mL/min/1.73 m^2^)	84.58 (11.3)
Bilirubin (µmol/L)	11.81 (5.01)
ALT (IU/L)	20.96 (10.26)
ALP (IU/L)	107.2 (57.28)

There was no difference in mean HbA1c after treatment between lixisenatide or placebo. There was no difference in mean BMI or body weight when comparing groups, before or after treatment. However while the body weight did not change after treatment with placebo, a small but significant reduction was noticed after lixisenatide (Table 2).

**TABLE 2:** Effect of treatment on HbA1C, BMI, and body weight as mean (SD)

	Lixisenatide	Placebo	*P* value
Baseline HbA1C (DCCT: %)	7.9 (0.5)	7.9 (0.5)	.99
HbA1C after treatment (DCCT: %)	8.1 (1.0)	8.0 (0.5)	.78
Baseline HbA1C (IFCC: mmol/mol)	63.8 (8.0)	63.9 (8.0)	.93
HbA1C after treatment (IFCC: mmol/mol)	64.7 (8.5)	64.1 (8.0)	.8
Difference in HbA1C (DCCT: %)	0.07 (0.3)	0.03 (0.4)	.58
Difference in HbA1C (IFCC: mmol/mol)	0.92 (3.1)	0.13 (4.0)	.44
Baseline BMI (kg/m^2^)	27.0 (3.5)	27.1 (3.5)	.98
BMI after treatment (kg/m^2^)	26.6 (3.6)	27.1 (3.5)	.61
Difference in BMI (kg/m^2^)	−0.48 (0.4)	−0.08 (0.5)	.001
Baseline body weight (kg)	78.8 (11.1)	79.0 (11.2)	.99
Body weight after treatment (kg)	77.6 (11.0)	79.2 (11.1)	.63
Difference in body weight (kg)	−1.4 (1.1)	1.1 (1.7)	<.001

### 3.2 Primary end‐point

**FIGURE 3:**
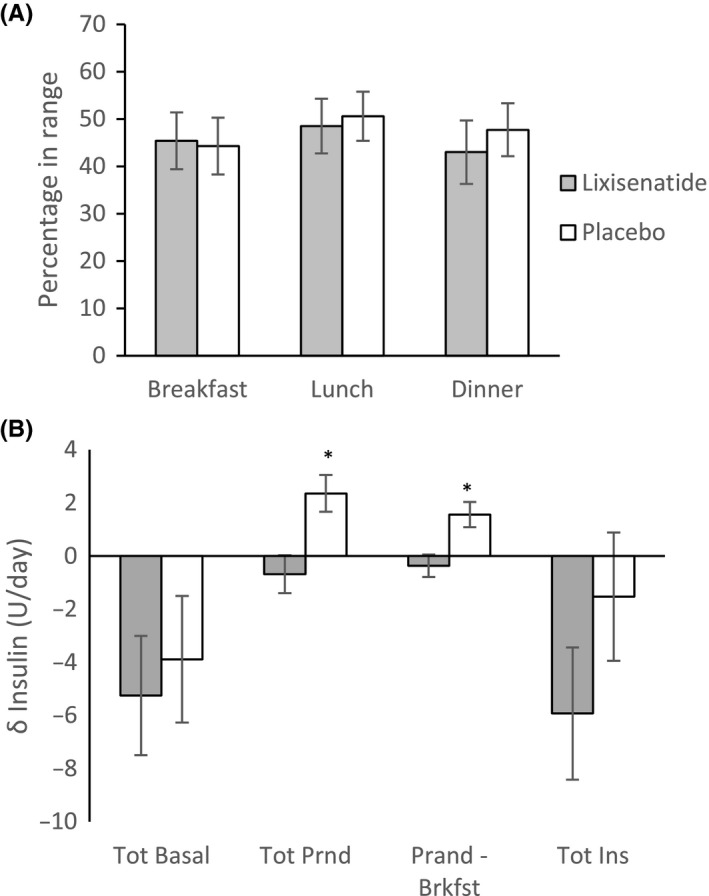
A, Percentage of CGM readings within 4‐10 mmol/L before and after treatment and between groups. B, Change in insulin dose between groups. Values in mean (SEM)

Continuous glucose monitoring data were analysed in all 25 data sets. After excluding incomplete data, comparison between groups pre‐treatment was between 25 data sets, while comparison between pre‐ and post‐treatment and between groups post‐treatment was in 22 data sets. The mean percentage of CGM readings in target range (4‐10 mmol/L) during the 3 hours after meals in range were similar before and after treatment, and between lixisenatide and placebo for all meals (Mean [SEM]: Breakfast 45.4 [6.0] % vs 44.3 [6.0] %, *P* = .9, lunch 45.5 [5.8] % vs 50.6 [5.3] %, *P* = .6 and dinner 43.0 [6.7] % vs 47.7 [5.6] %, *P* = .6; Figure 3A, Table S1).

### 3.3 Insulin requirement

There was a numerically lower total basal insulin dose after treatment in both groups, with a greater reduction seen after lixisenatide (26.6 [2.8] U/d to 21.4 [1.9] U/d after lixisenatide, *P* = .4, and 27.2 [3.0] U/d to 23.3 [2.3] U/d after placebo, *P* = .6); however, this was not significant between groups before or after treatment (Figure 3B, Table S1). The total prandial insulin was numerically lower after treatment with lixisenatide (19.0 [3.0] U/d to 18.4 [2.9] U/d, *P* = .1) and numerically higher after treatment with placebo (18.3 [2.9] U/d to 20.5 [3.0] U/d, *P* = .06), resulting in a significantly lower total prandial insulin dose after treatment with lixisenatide compared with placebo. This drop in prandial insulin dose with treatment was most prominent after breakfast (Figure 3B, Table S1).

### 3.4 Mixed meal test

**FIGURE 4:**
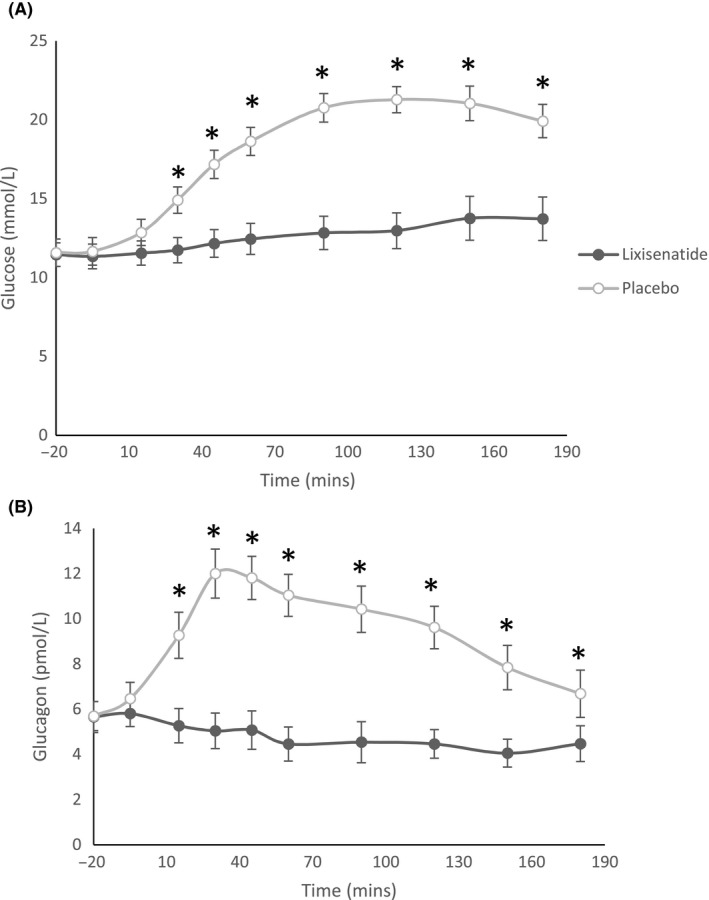
A, Blood glucose level after standardised mixed meal. B, Blood glucagon level after standardised mixed meal. Values in mean (SEM)

All 25 patients participated in the meal tolerance test for 120 minutes, and 18 of them had the test extended by another hour to 180 minutes. Mean plasma glucose level after the lixisenatide treatment period expressed as Mean (SEM) was 11.2 (0.8) mmol/L at baseline, increasing to 13.0 (1.0) mmol/L at 120 minutes and 13.6 (1.4) mmol/L at 180 minutes. Glucose levels after placebo were 11.5 (0.9) mmol/L at baseline (*P* = .48 vs lixisenatide) and increased at 120 minutes to 21.3 (0.7) mmol/L (*P* < .001) and at 180 minutes to 20.0 (1.1) mmol/L (*P* = .001). Corresponding glucagon level after lixisenatide at baseline was 5.8 (0.7) pmol/L and reduced at 120 minutes to 4.5 (0.5) pmol/L, and at 180 minutes to 4.5 (0.8) pmol/L. In contrast, glucagon level after placebo was 6.5 (0.6) pmol/L (*P* = .44 vs lixisenatide) at baseline and increased at 120 minutes to 9.5 (0.8) pmol/L (*P* < .001) and then settled at 180 minutes to 6.7 (1.0) pmol/L (*P* = .09) (Figure 4A, Table S2). The AUC at 120 minutes for glucose was 392.0 (167.7) mmol/L × min after lixisenatide and 628.1 (132.5) mmol/L × min after placebo (*P* < .001). The AUC at 120 minutes for glucagon was 140.0 (110.0) nmol/L × min after lixisenatide and 304.2 (148.2) nmol/L × min after placebo (*P* < .001) (Figure 4, Table S2).

### 3.5 Hyperinsulinaemic hypoglycaemic clamp

**FIGURE 5:**
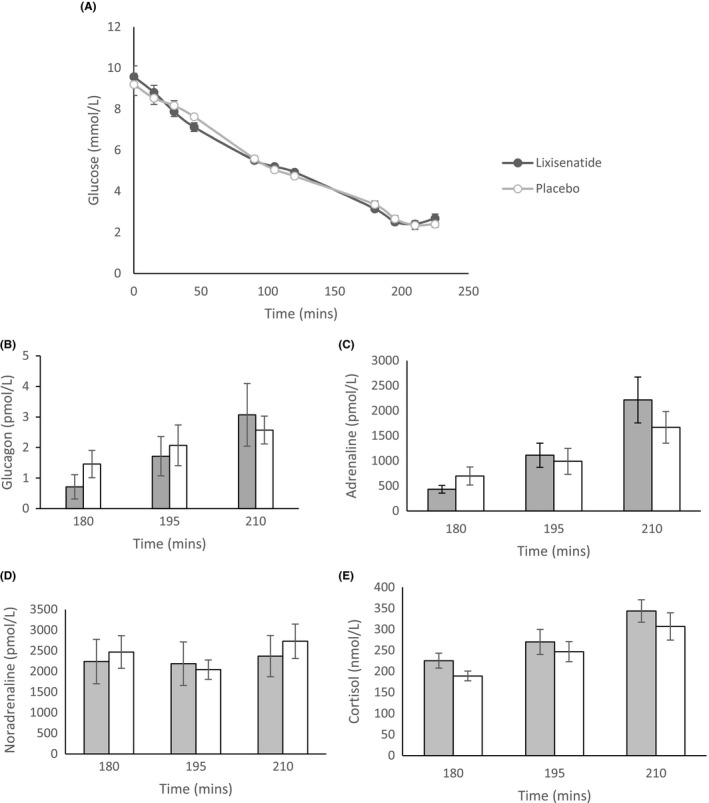
A, Blood glucose level during hyperinsulinaemic hypoglycaemic clamp between groups. B‐E, Counter‐regulatory hormone levels during the hypoglycaemic phase between groups. Values in mean (SEM)

A total of 15 patients agreed to undergo the hypoglycaemic clamps, but one individual only completed one clamp and so this data was excluded, leaving 14 paired clamps to be analysed. Mean plasma glucose level at the start of the clamp was the same between groups, and fell steadily during the clamp in both groups to euglycaemic level between 90 to 135 minutes (Figure 5A, Table S3). Hypoglycaemic threshold was reached between 180 and 225 minutes in both groups. Glucagon level was found to fall in both groups up to 180 minutes and then increased during hypoglycaemic phase (180‐225 minutes) of the clamp. There was no significant difference in glucagon level during hypoglycaemia between groups. Other counter‐regulatory hormones—adrenaline, noradrenaline, cortisol and pancreatic polypeptide (PP) increased during hypoglycaemia, but there was no significant difference between groups (Figure 5B‐E, Table S3).

### 3.6 Adverse events

A total of 840 adverse events were reported in 27 patients during the study (Table 3). One serious adverse event was reported during treatment with placebo, when loss of consciousness resulted in hospital attendance. Blood glucose testing at site confirmed that this was not related to hypoglycaemia. None of the adverse effects resulted in withdrawal from the study. Another participant became pregnant during treatment with placebo did not enter the lixisenatide arm and was excluded from the study.

**TABLE 3:** Number of adverse events reported by patients during 4 wk of treatment compared to the week prior to the treatment

Treatment	Hypoglycaemia	Gastrointestinal side effects	Headache	Diabetes eye complications	Infections	Loss of consciousness	Pregnancy	Nonspecific	Total
Lixisenatide	298	12	5	1	2	0	0	2	320
Placebo	421	2	1	2	5	1	1	3	436
Pretreatment	84	0	0	0	0	0	0	0	84

The majority of the adverse events (95% of total) reported by participants related to hypoglycaemia (defined as capillary blood glucose <4 mmol/L). Reported hypoglycaemic episodes in the prerandomisation period were 84 accounting for 3.2 episodes per patient per week, 298 episodes during treatment with lixisenatide (2.9 episodes per patient per week) and 421 during treatment with placebo (4.1 episodes per patient per week). There were no reported episodes of severe hypoglycaemia.

Gastrointestinal side effects (nausea, vomiting, bloating and diarrhoea) were the next most commonly reported side effect, with 12 episodes were reported with lixisenatide and 2 episodes with placebo. Other common side effects were headache and minor infections. There was no incidence of Diabetic Keto Acidosis during the trial.

## 4 DISCUSSION

In this first study with lixisenatide in patients with T1D, 4‐week exposure to lixisenatide did not make significant difference to the prespecified primary end‐point of proportion of CGM readings within the defined range of 4‐10 mmol/L in the post‐prandial period. The dose of insulin was reduced at the start of treatment, and patients were advised to titrate the dose up to maintain their blood glucose level between 6 and 9 mmol/L. It was noticed that significantly less insulin was being used at the end of treatment period with lixisenatide to achieve a similar time in range after meals. As expected the insulin dose could be reduced most at breakfast and did not change with the evening meal when the effect of lixisenatide may have declined. Although patients only had treatment for 4 weeks, there was significant reduction in mean body weight after treatment with lixisenatide.

The most striking findings from our study were seen in the MMT, where lixisenatide was given, but not rapid insulin. We observed a large attenuation in glucose rise, accompanied by suppression of glucagon compared with the placebo group in the absence of prandial insulin. This reduction in PPBG was not seen during the 4‐week treatment period, and we hypothesise that it is likely that most patients did not have enough time within the 4 weeks to fully intensify insulin treatment to achieve their usual glycaemic goals. This could explain why the proportion of post‐prandial blood glucose levels in the euglycaemic range was comparable between groups, despite a lower prandial insulin dose. This could be tested in trials with longer exposure to lixisenatide. The effect of lixisenatide on slowing down gastric emptying may significantly contribute to its effect on PPBG. This is less likely to be seen in longer‐acting GLP1 analogues like liraglutide as the effect on gastric emptying may be blunted over time, owing to tachyphylaxis. This study did not examine gastric emptying with lixisenatide.

The clamp study also demonstrated that although glucagon level was reduced in the post‐prandial period, there was no significant effect on counter‐regulatory hormone level during hypoglycaemia during treatment with lixisenatide. The safety of lixisenatide in T1D patients was established, and there was less patient reported hypoglycaemia during treatment with lixisenatide than placebo. In line with other trials involving GLP1 receptor agonists, there was higher incidence of gastrointestinal side effects during treatment with lixisenatide compared with placebo.

Our findings are consistent with previous studies investigating the effect of GLP1 receptor agonist liraglutide in people with T1D.^12^ In the first trial with liraglutide, 4‐week treatment did not make any difference to glucose levels, although reduced insulin requirement was observed. In longer trials, liraglutide was found to result in small but significant improvement in HbA1C level after 12, 26 and 52 weeks treatment.^13^, ^14^, ^15^ In our trial, similar to liraglutide,^12^ the dose of insulin was significantly reduced at the start of the treatment to avoid risk of hypoglycaemia.

This study comprehensively investigates the effect of lixisenatide on post‐prandial blood glucose in a real life setting, as well as experimental conditions.

In summary, our study raises the possibility that in selected patients, a short‐acting GLP‐1 receptor agonist could be a useful adjunctive treatment in T1D to limit post‐prandial glucose rise.

## CONFLICT OF INTEREST

The authors have no conflict of interest to declare.

## AUTHOR CONTRIBUTIONS

Chitrabhanu Ballav conceived and designed the study under supervision of Prof SCL Gough (see above), recruited and consented subjects, performed the experiments (CGM, Mixed Meal Test, and Clamp), analysed the data and drafted the manuscript. Archana Dhere recruited and consented subjects, performed the experiments and analysed the data. Irene Kennedy helped with analysis of data and contributed to drafting the manuscript. Olorunsola Agbaje performed the statistical analysis on primary end‐point and contributed to drafting the manuscript. Sarah White and Rachel Franklin performed key study visits, performed the experiments, and contributed to drafting the manuscript. Bolette Hartmann analysed samples from the study and contributed to drafting the manuscript. Jens J. Holst offered advice on designing the study, analysed samples from the study and contributed to drafting the manuscript. Rury R. Holman offered advice on designing the study, had oversight on performing the study and analysis of results and contributed to drafting the manuscript. Katharine R. Owen was the head oversight over designing and performing the study, analysis of the results and supervised the drafting of the manuscript.

## ETHICAL APPROVAL

The study was performed in accordance with good clinical practice and was approved by the clinical ethics committee in UK (ISRCTN No. 00290196). Patients were recruited from outpatient clinics at the Oxford Centre for Diabetes, Endocrinology and Metabolism. Informed consent was obtained prior to participation, and the study was monitored by an external monitor.

## Supporting information

Table S1‐S3

## Data Availability

Individual participant data will not be available.
