# Supplementary material for: Lixisenatide in type 1 diabetes: A randomised control trial of the effect of lixisenatide on post‐meal glucose excursions and glucagon in type 1 diabetes patients
Source: Endocrinol Diabetes Metab. 2020 Jun 12;3(3):e00130. doi: 10.1002/edm2.130 (PMC7375047; doi:10.1002/edm2.130)
Supplement: Supplementary file 1 — Table S1‐S3 [file EDM2-3-e00130-s001.docx]

**Contents**

CGM supplementary table 2

Meal Tolerance Test supplementary table 3

Clamp supplementary table 4

Supplementary table 1. CGM glucose level in mmol/L in Mean (SEM).

Supplementary table 2. Meal Tolerance Test glucose and glucagon levels in Mean (SEM).

Supplementary table 3. Hyperinsulinaemic hypoglycaemic clamp data showing counter-regulatory hormone level during hypoglycaemia in Mean (SEM).
